# Treating Tet Spells With Disopyramide in a 72-Year-Old Awaiting Primary Repair of Tetralogy of Fallot

**DOI:** 10.1016/j.jaccas.2023.102093

**Published:** 2023-11-03

**Authors:** George T. Kalapurakal, James M. Monaco

**Affiliations:** Department of Cardiology, Advanced Heart Failure, Advocate Christ Medical Center, Chicago, Illinois, USA

**Keywords:** disopyramide, tet spells, tetralogy of Fallot

## Abstract

An adult with unrepaired tetralogy of Fallot presented with frequent tet spells. Her course was complicated by severe cyanotic spells and tachycardia-bradycardia syndrome that limited beta blocker use to stabilize her spells. She markedly improved after disopyramide initiation and underwent successful tetralogy of Fallot repair with excellent functional outcome.

## Graphical abstract

**Figure:**
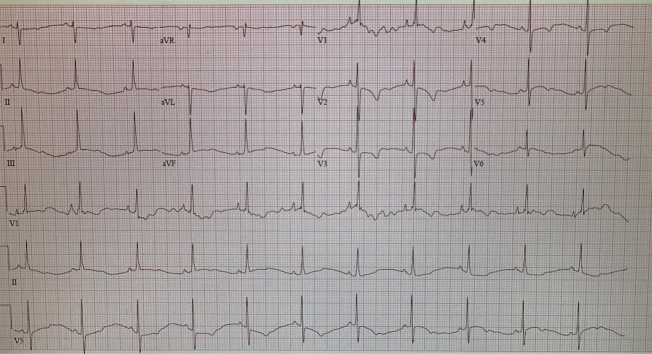

## History of Presentation

A 72-year-old woman with notable past medical history of unrepaired tetralogy of Fallot (TOF) was transferred for management after stabilization at an outside hospital.

**Learning Objectives**

• To describe one of the oldest reported patients with unrepaired TOF.
• To describe treatment strategies in TOF.
• To recognize the role of disopyramide to relieve hypercyanotic spells when patients cannot tolerate typical treatment regimens.

For years, the patient had reportedly experienced seizure-like episodes associated with activity, during which she would tense her body for about 15 minutes and noted shortness of breath, abdominal discomfort, and dusky lips. However, she remained able to interact, with no confusion or lethargy. These episodes occurred with increasing frequency leading up to presentation.

During her initial admission, she was treated for possible abdominal sepsis with fluid boluses and antibiotics. Laboratory test results were notable for white blood cell count of 21,000/μL, bicarbonate level of 15 mmol/L, and lactic acid level of 8.3 mmol/L. Although she initially improved, she experienced recurrent spells of severe hypoxemia and visible cyanosis, prompting her transfer to our center for cardiac surgical evaluation. On arrival, vital signs were notable for oxygen saturation of 88% on room air. Physical examination revealed a 2/6 systolic ejection murmur, a left lateral thoracotomy scar, equal pulses in the extremities, and an absence of clubbing of her fingernails. Previously noted abnormalities in blood work had resolved. Shortly after admission, she experienced a spell with severe cyanosis with oxygen saturation as low as 29%, during which the patient tensed her body and brought her knees to her chest. She remained conscious and interactive throughout, with normal recall of events afterward. The spell resolved after administration of supplemental oxygen, intravenous phenylephrine, a fluid bolus, and intravenous metoprolol.

## Past Medical History

The patient’s medical history included TOF with unsuccessful attempt at surgical palliation in India during childhood, hypothyroidism, and reported seizure disorder.

## Differential Diagnosis

The differential diagnosis included seizure, sepsis, and worsening of the severity and frequency of cyanotic episodes in a patient with unrepaired TOF.

## Investigations

**Figure 1:**
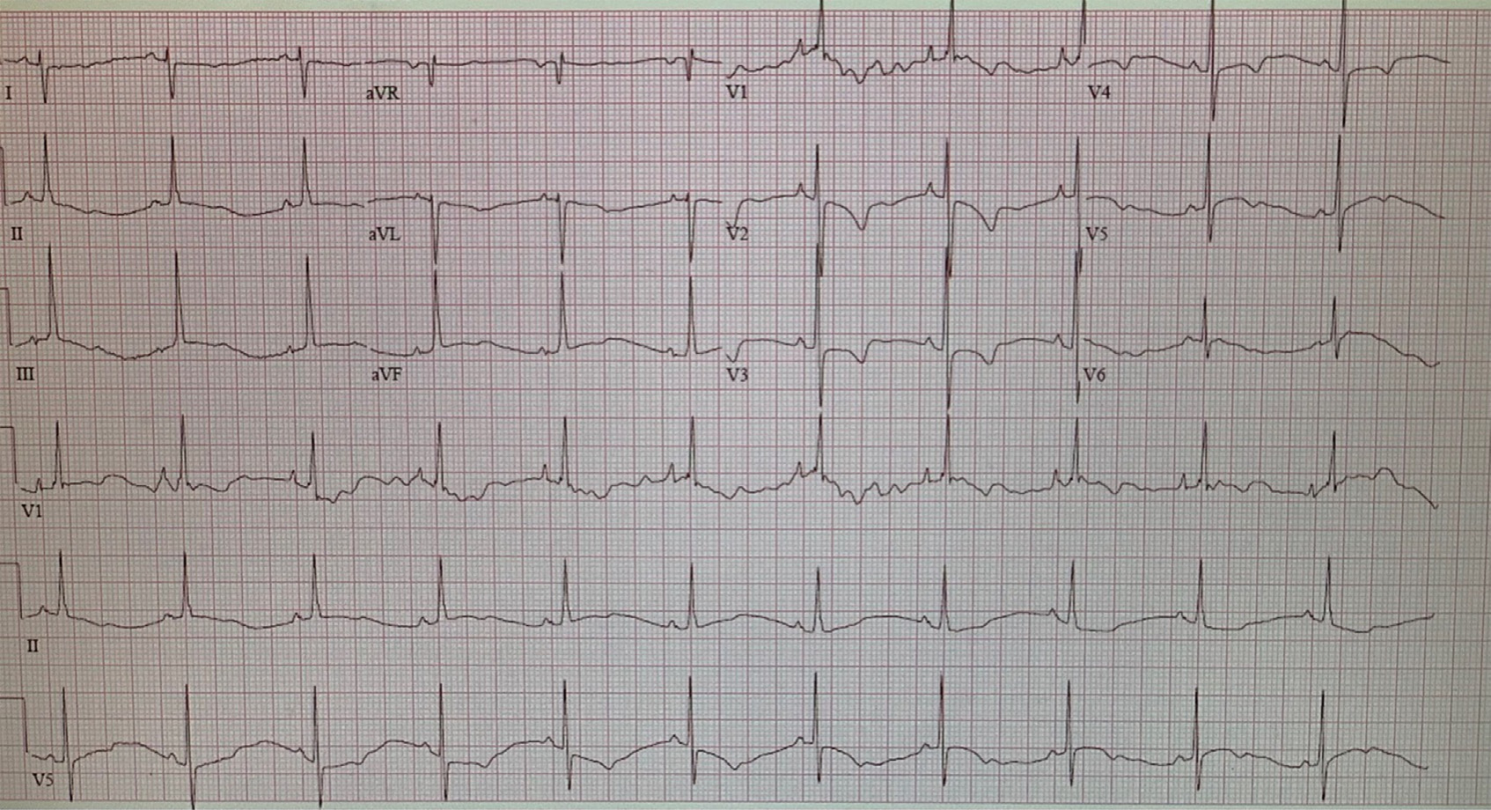
Electrocardiogram Normal sinus rhythm with right ventricular hypertrophy and ST-/T-wave changes.

Electrocardiogram (Figure 1) demonstrated normal sinus rhythm with right ventricular (RV) hypertrophy and ST-/T-wave changes in the anterolateral leads.

**Figure 2:**
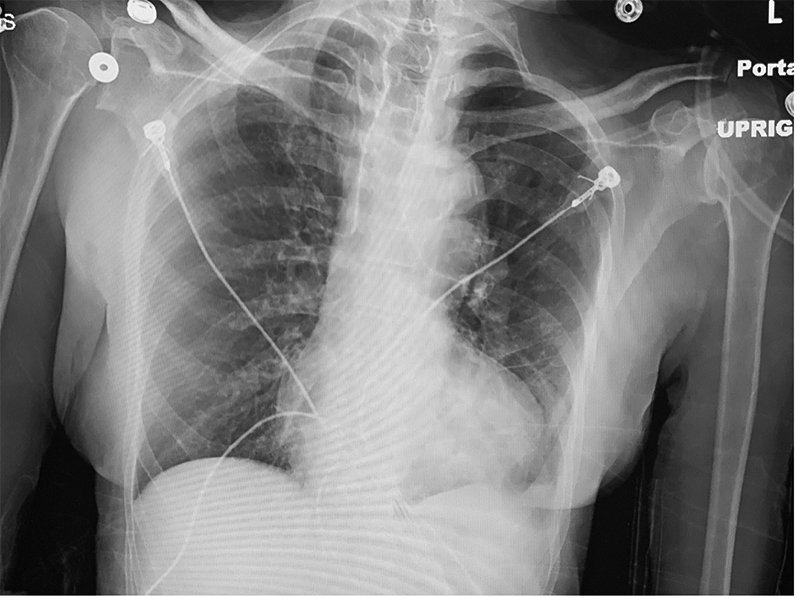
Chest X-Ray Film No acute pulmonary pathology.

X-ray (Figure 2) and nongated computed tomography imaging of the chest demonstrated no pulmonary pathology. Transthoracic echocardiograms were obtained on admission and hospital day 5 and demonstrated normal left ventricle size and systolic function as well as findings consistent with unrepaired TOF: a large subaortic perimembranous ventricular septal defect (VSD) with low-velocity, bidirectional flow; overriding aorta with moderate root dilation with no obvious regurgitation; and a mildly dilated and severely hypertrophied RV with severe, dynamic outflow tract stenosis with a peak gradient of 70 to 90 mm Hg.

Cardiac computed tomography scan demonstrated unrepaired TOF consisting of large anterior conal malalignment VSD with overriding aorta, RV hypertrophy and multilevel severe RVOT obstruction, predominantly subvalvular but with dysplastic bicuspid pulmonary valve and moderate supravalvular narrowing. No significant obstruction or hypoplasia was present in the distal pulmonary vasculature. No patent aortopulmonary shunt was identified.

## Management

Her course was notable for recurrent stereotyped episodes, which were determined to be tet spells. Ongoing use of phenylephrine to maintain mean arterial pressure over 80 mm Hg, intravenous fluid administration, and supplementary oxygen by nasal cannula ameliorated their severity but did not prevent frequent cyanosis. Right heart catheterization was deemed high risk for iatrogenic RVOT muscle spasm inducing a severe tet spell and, hence, was not performed. Additionally, noninvasive imaging confirmed the RVOT gradient and thereby low likelihood for pulmonary arterial hypertension. Oral metoprolol tartrate was initiated to reduce RV inotropy and thus minimize dynamic RVOT obstruction.

Telemetry subsequently demonstrated sinus bradycardia with heart rates of 35 to 45 beats/min with isolated monomorphic ectopy. When metoprolol was then held, the patient developed frequent nonsustained narrow complex tachycardias. Given a need to reduce RV inotropy and suppress atrial tachyarrhythmias without inducing bradycardia, disopyramide was initiated at a dose of 100 mg every 6 hours with successful resolution of cyanosis, suppressions of ectopy, and maintenance of normal sinus rhythm.

Once stabilized, the patient underwent successful TOF repair with transvalvular RVOT patch, pulmonary valve replacement with a bioprosthetic valve, and resection of RVOT muscle bundles.

## Discussion

TOF is the most common cyanotic congenital heart anomaly. The underlying developmental defect is abnormal septation of the great arteries and malalignment of the conal septum, which produces the classic syndrome of a VSD, overriding aorta, multilevel RV outflow obstruction, and RV hypertrophy.^1^ Modern management calls for primary intracardiac surgical repair as early as 1 to 2 months of age. Although associated with excellent long-term outcomes, the lifetime risks of cardiac morbidity and mortality remain higher than the general population.

Unfortunately for patients unable to receive surgical repair in childhood, survival rates are poor, with some studies estimating that approximately only 3% reach their fourth decade of life.^2^ Long-term survival in this population requires that the obstruction to pulmonary flow remain in a precarious balance because excessive pulmonary obstruction will result in a fatal degree of cyanosis, whereas too little obstruction will fail to protect the pulmonary vasculature from pressure and volume overload, leading to pulmonary arterial hypertension and Eisenmenger syndrome. For those with a significant dynamic RVOT component, such as our patient, obstructive episodes often manifest themselves as “tet spells,” where patients engage in behaviors that raise systemic vascular resistance to reduce right to left shunting, like Valsalva and bringing the knees to the chest.^3^ There are limited case reports in the modern era documenting late survival without surgery, with our patient being one of the oldest to receive repair.

Inpatient management of these spells includes raising the systemic vascular resistance with vasopressors like phenylephrine, administering intravenous fluids to improve RV filling and reduce dynamic RVOT collapse, providing oxygen to serve as a pulmonary vasodilator, and administering intravenous beta blockers or calcium-channel blockers to reduce inotropy and infundibular spasm. Collectively, these interventions reduce obstruction in the RVOT and right-to-left shunting across the VSD, leading to improved pulmonary circulation.^4^

Clinically significant arrhythmias are another major source of morbidity in TOF, both repaired and unrepaired.^5^^,^^6^ Our patient suffered from tachycardia-bradycardia syndrome, which complicated management by preventing the use of metoprolol. We therefore tried to manage rhythm and dynamic outflow obstruction with the Class Ia antiarrhythmic disopyramide. Like its benefit in hypertrophic obstructive cardiomyopathy because of its negative inotropic action, disopyramide can alleviate dynamic RVOT obstruction in TOF patients.^7^ QT prolongation and arrhythmias are reported complications that should be monitored for^8^; however, our patient tolerated disopyramide, well with suppression of hypercyanotic spells and arrhythmias until surgical repair.

## Follow-Up

Our patient tolerated TOF surgical repair well with an uncomplicated postoperative inpatient course. Transthoracic echocardiogram obtained 1 month after surgery demonstrated mild RV hypertrophy with unobstructed RVOT and normal RV pressures, a well-functioning bioprosthetic pulmonary valve, and trivial inferior ventricular patch leak. There was severe septal hypokinesis consistent with the presence of a VSD patch contributing to overall low-normal left ventricular systolic function.

## Conclusions

Current management of TOF in resource-rich health care systems calls for primary surgical repair performed in infancy. However, patients born without immediate access to modern diagnostic and surgical techniques may be left with the condition unrepaired or only partially palliated into adulthood. Management of such patients requires medically stabilizing cyanotic spells via treating dynamic RVOT obstruction and assessing eligibility for late repair. Our patient’s unique case demonstrates the utility and safety of the antiarrhythmic and negative inotropic agent disopyramide in relieving dynamic RVOT obstruction as well as the feasibility and survivability of intracardiac repair of TOF even in a patient in her eighth decade of life.

## Funding Support and Author Disclosures

The authors have reported that they have no relationships relevant to the contents of this paper to disclose.
